# Deep feature optimization using fusion of multiple self-supervised learning approaches and filter-based feature selection for lung cancer histopathology classification

**DOI:** 10.1371/journal.pone.0348194

**Published:** 2026-04-27

**Authors:** Nepolian Vailankanni, Bharanidharan Nagarajan

**Affiliations:** School of Computer Science Engineering and Information Systems, Vellore Institute of Technology, Vellore, India; Public Library of Science, UNITED KINGDOM OF GREAT BRITAIN AND NORTHERN IRELAND

## Abstract

One of the most deadly illnesses in the world is lung cancer, and increasing survival rates require early detection. Lung cancer diagnostics from the imaging modalities is always subjective, and this paves the way for deep learning assisted computer aided techniques. Still, the accuracy of such a technique is the major concern. This research work attempts to enhance lung cancer diagnostics from histopathological images using a previously unaddressed combination of multiple self-supervised learning techniques, filter-based feature selection, and Vision Graph Convolutional Networks. The main contribution lies in optimized feature fusion, and it brings complementary strengths of three different self-supervised learning approaches- contrastive alignment, redundancy reduction, and semantic grouping. The first step involves extracting the key features from histopathological images using a custom Convolutional Neural Network. Three complementary self-supervised learning methods – Deep Cluster, Bootstrap Your Own Latent, and Simple Framework for Contrastive Learning of Visual Representations – are then used to refine each of these features separately. Following optimization, the improved features are combined, and the most significant features from the combined set are found and preserved using a filter-based feature selection technique called Minimum Redundancy Maximum Relevance. Vision Graph Convolutional Neural Network is used as a classifier. Initial experiments are carried out on the 691 histopathological images extracted from the publicly available LungHist700 dataset. This dataset contains histopathological images under three categories: 151 normal subjects, 280 lung adenocarcinoma subjects, and 260 lung squamous cell carcinoma subjects. The proposed approach provided a balanced accuracy of 97% while the plain Vision Graph Convolution Neural Network offers only an 86% balanced accuracy score. Further validation is performed using two more publicly available datasets, namely LC25000 and TCGA UT datasets. The experimental results demonstrate the enhanced lung cancer prediction performance of the proposed approach in all three datasets.

## Introduction

Cancer continues to be one of the leading causes of death worldwide, primarily due to its uncontrolled cell growth, which can invade nearby tissues and spread to distant organs. Among all cancer types, lung cancer accounts for the highest number of cancer-related deaths, largely because of its aggressive progression and the fact that it is often detected at an advanced stage [1]. The World Health Organization estimates that in 2022 alone, there were about 1.8 million lung cancer deaths, accounting for 18.7% of all cancer-related deaths, and an estimated 2.5 million new cases, or 12.4% of all newly diagnosed cancers [2]. This alarming trend highlights how urgently trustworthy and efficient diagnostic methods are needed to enhance lung cancer management and treatment. Radiological and histological methods like computed tomography, X-rays, magnetic resonance imaging, histopathological examination, and positron emission tomography are used in current diagnostic procedures [3]. A CT scan is helpful in lung cancer diagnosis, staging, and monitoring. When compared to a typical chest X-ray, a CT scan offers fine-grained cross-sectional images of the lungs, making it possible to more accurately identify masses, nodules, lymph node involvement, and metastatic spread [4]. MRI provides greater soft-tissue differentiation, and it is used to assess mediastinal invasion or the chest wall [5]. PET combines structural and metabolic imaging, and it is vital for determining distant metastases, staging, and evaluating the effectiveness of treatment [6]. Despite their benefits, these imaging methods are constrained by their incapacity to categorize tumour subtypes with certainty and grade tumours according to cellular morphology. On the other hand, the definitive method for conclusive prediction of lung cancer and subtyping is biopsy histopathological examination. By providing cellular and subcellular resolution, histopathology helps pathologists spot important morphological characteristics such as architectural patterns, mitotic activity, and nuclear pleomorphism [7].

Lung cancer can be broadly divided into two groups: non-small cell lung cancer, which makes up nearly 85% of cases, and small cell lung cancer, which makes up 15% of cases [8]. The aggressive neuroendocrine nature, fast growth, early metastasis, frequent relapse, and high resistance to treatment are characteristics of small-cell lung cancer. With a five-year survival rate of less than 10%, the prognosis is still poor despite treatment advancements, highlighting the critical need for more precise prognostic instruments and individualized treatment plans [9]. Adenocarcinoma, squamous cell carcinoma, and large cell carcinoma are among the subtypes of non-small cell lung cancer that have different morphological and molecular traits.

Detecting lung cancer in an earlier stage is still very difficult, even with major advancements in diagnostic imaging and treatment approaches [10]. A major problem is that most patients receive their diagnosis at a late stage; more than 50% of cases of lung cancer are discovered only when the disease has progressed, primarily because early disease is asymptomatic [11]. Hence, accurate diagnostics are the key to handling lung cancer. Manual histopathological analysis is frequently labour-intensive, subjective, and prone to discrepancies between observers. New paths for automated histopathology–based decision support systems have been made possible by recent advancements in digital pathology and artificial intelligence, enabling more reliable and scalable diagnostic assistance [12].

Clinical decision-making and medical image analysis have been transformed by deep learning, a subfield of artificial intelligence, particularly in the precise identification and diagnosis of cancer. It learns data representations directly from input features using multi-layered neural network topologies, eliminating the need for manual engineering. Convolutional Neural Networks, among the most widely used deep learning architectures, have demonstrated outstanding performance in image classification, object detection, and segmentation tasks, owing to their ability to capture and represent spatial hierarchies within data [13]. The ability of Recurrent Neural Networks to model temporal dependencies through internal memory makes them popular for use in the analysis of sequential and time-series data. This makes them particularly helpful for healthcare tasks like patient monitoring, disease progression modelling, and diagnostic prediction [14]. Graph Neural Networks (GNN), a potent DL framework for simulating intricate interactions between non-Euclidean data points, such as tissue areas, cellular morphology, or patient similarity graphs. GNNs make it possible to include structural, geographical, or semantic linkages into learning, and they have shown great efficacy in biological settings [15]. Self-Supervised Learning (SSL) falls under the DL category, and even in situations where there is a limited quantity of labeled data available, it is frequently used to extract significant feature patterns from unlabeled data and then modify them for particular tasks. An SSL technique called self-pretraining makes use of a carefully selected dataset to pretrain and fine-tune the networks [16]. SSL improves downstream classification performance in low-label scenarios while simultaneously lowering annotation expenses. SSL has demonstrated encouraging outcomes in pathology and radiology when paired with GNN and CNN, enabling reliable feature extraction and enhancing the generalization of diagnostic models [17].

Medical diagnosis is one of the primary applications of deep learning. This covers biomedicine, radiographic image analysis, and health informatics, among other fields [18]. DL is being investigated for multi-modal diagnostics in addition to imaging, combining information from genomic sequences, clinical data, and radiological images to produce more reliable clinical insights [19]. The ability of DL to match or even surpass expert-level performance in particular diagnostic tasks is one of the main factors driving its adoption in the healthcare industry. Clinical implementation still faces obstacles despite its potential, such as issues with interpretability, data protection, and the generalizability of DL models across institutions [20]. However, as annotated medical datasets become more widely available and DL techniques advance quickly, these models are increasingly being incorporated into clinical workflows and computer-aided diagnosis systems [21].

The prime objective of this work is to enhance deep features for improving the lung cancer classification performance using histopathological images. The proposed framework leverages a unique integration of multiple SSL, filter-based feature selection, and graph-based modelling, which has not been extensively explored previously. Three SSL techniques, namely Simple framework for Contrastive Learning of visual Representations (SimCLR), Deep Cluster, and Bootstrap Your Own Latent (BYOL), are employed to enhance features. Then, a filter-based feature selection technique, Minimum Redundancy Maximum Relevance (mRMR), is implemented to select the important feature subset from the enhanced feature set. Finally, Vision Graph Convolutional Neural Network (VGCN), a type of GNN, is used as a classifier.

The main contribution of this work is itemised as follows:

1. A novel DL pipeline that fuses custom CNN extracted features from three complementary SSL approaches – SimCLR (contrastive), BYOL (non-contrastive), and DeepCluster (clustering-based), and their fusion brings complementary strengths – contrastive alignment, redundancy reduction, and semantic grouping. It leads to feature optimization through richer and more diverse feature representations.
2. Usage of the mRMR technique to select the most relevant and non-redundant features from the high-dimensional fused SSL representations. Its ability to effectively handle diverse feature sources makes it particularly well-suited for refining multi-SSL outputs.
3. Implementation of VGCN to model complex spatial relationships among the selected features from the fused SSL representations and enhance the structural understanding of histopathological patterns, thereby providing better performance in detecting lung cancer.

The remainder of the paper is organized as follows: The next section elaborates on the related works, and the third section deals with the background of the algorithms used; the proposed methodology is discussed in the fourth section, and the Results & discussion are presented in the fifth section. The conclusion is presented in the last section.

## Related Works

Research on lung cancer detection has been ongoing, and multiple studies have found different approaches for improvements. This section examines current developments in the classification of lung cancer, emphasizing significant contributions and breakthroughs in the field.

Traditional Machine Learning (ML) algorithms like Support Vector Machines, Random Forest, and Decision Trees were used extensively in early lung cancer detection research for tasks including nodule identification and categorization. These models’ success was heavily reliant on feature quality, necessitating feature engineering expertise [22]. Deep Learning, particularly CNN, which can automatically learn hierarchical representations from raw images, addressed the flaws of ML techniques. CNN-based models have shown cutting-edge results in the analysis of histopathological images for lung cancer subtyping and radiology imaging, including CT, MRI, and PET scans. For example, a CNN variant – InceptionV3 is utilized to perform categorization of non-small cell lung cancer subtypes [12]. In another work, SqueezeNet, which is a lightweight CNN, was implemented in lung CT scan feature extraction, and the extracted features are classified using an ML technique, namely Logistic regression [23].

GNN is a relatively recent development that models the intricate spatial interactions between tissue components in histopathology images. When compared to GNN, Graph Convolutional Network (GCN) is a ground-breaking DL architecture that adapts traditional convolutional neural networks to non-Euclidean domains, which makes them perfect for organized biomedical data [24]. For instance, a graph-based multiple instance learning framework performed better in classifying lung cancer subgroups in the TCGA dataset. In their work, whole-slide image patches were represented as graph nodes, and inter-patch similarity was captured using a learned adjacency matrix [25]. Ahmedt Aristizabal D et. al. demonstrated the computational histopathology’s use of graph-based deep learning and presented how graphs constructed from cell, tissue area, or patch features outperform CNN-only techniques in tumor identification [26]. Ram et al. proposed a graph-based sparse Principal Component Analysis method for lung cancer detection from histopathology images, emphasizing the ability of graph structures to preserve spatial and morphological dependencies between image regions [27]. Similarly, Parisot et al. introduced a graph-based framework using GCN to detect autism spectrum disorder and Alzheimer’s disease. Subjects were modelled as nodes with imaging features, and edges were based on non-imaging similarities. This work is evaluated on ABIDE and ADNI datasets; the method achieved 70.4% and 80.0% accuracy, respectively, demonstrating the effectiveness of GCNs in integrating multimodal data for medical diagnosis [28].

SSL is a potent way to learn relevant representations on unlabelled data, notably in fields in which labelled data is small or expensive to collect. Wei et al. proposed an autonomous-based GNN framework that can extract rich network semantics of the heterogeneous information networks. They used dual-channel contrastive learning to execute their method to advance the discriminative ability of node representations without engaging in manual labelling [29]. Yao and Yang addressed the challenges of feature degradation in low-shot video object detection by developing a spatial–temporal SSL framework. They incorporated a temporal continuity constraint to guide the learning of stable object features across frames [30]. Pan et al. proposed a self-supervised feature enhancement network for learning of classes that were incremental, i.e., in scenarios when the number of classes was small. The architecture used a two-encoder approach to learn both class and general representation, and contrastive alignment was strengthened further [31]. To detect the small objects in the noisy environment, Lee et al. use an improved network architecture with an SSL. Their approach focused on noise-robust feature maps, and these maps were learned through an auxiliary denoising autoencoder that supervised the feature backbone with a reconstruction loss [32]. Vilabella et al. introduced a new object detection structure, with an SSL foundation, assessed on the general visualist end-use. Their method is to propose a masked region prediction network, along with self-distillation to construct semantically dense representations of raw visual images [33]. Rahman et al. assessed the utility of self-supervised feature learning in digital pathology, comparing it with traditional transfer learning and emerging foundation models. Their study introduced a pathology-specific SSL pipeline trained on histopathological slides using a pretext task of tissue region prediction [34].

Overall, these works affirm the adaptability and promise of SSL in refining feature representations in many disciplines. Although different SSL approaches and handcrafted feature selection techniques for histopathological image analysis have been studied in the literature, the majority of these approaches concentrate on a single SSL framework or fail to consider the synergy between various SSL representations. Additionally, especially in the context of multi-class lung tumour classification, little focus was placed on incorporating optimized feature selection techniques that can reduce high-dimensional features to the most discriminative ones. On the other hand, the ability of GCN and GNN variants to model intricate structural patterns and capture non-Euclidean relationships makes them especially effective for analysing data that has contextual or spatial dependencies, like histopathological tissue structures. But the integration of VGCN with optimized deep features for histopathological classification is still largely unexplored, despite recent studies introducing them to model spatial dependencies in image data. This leaves a research gap in the development of strong, broadly applicable frameworks that can provide robust and generalized solutions in the classification of histopathological patterns. By putting forward a unified deep feature optimization pipeline that combines complementary features from various SSL approaches, employing a filter-based selection strategy to preserve the most informative representations, and by implementing the efficient VGCN architecture as a classifier, we overcome this limitation in this work.

## Background

### Graph convolutional networks

Graph Convolutional Networks generate node representations by aggregating information across multiple convolutional layers. In this process, they make use of both the underlying graph structure and the associated input features to effectively learn meaningful embeddings. The structural information in the neighbourhood of a node’s l-hop network is recorded by its representation after l aggregation iterations. GNN’s l-th layer is mathematically represented using Equation (1).


$$\;\overset{\left(l\right)}{\underset{n}{q}}={AGGREGATE}^{\left(l\right)}\left(\{\overset{\left(l-\text{1}\right)}{\underset{u}{x}}:u\in N(n)\}\right)$$ (1)

where $\overset{\left(l\right)}{\underset{n}{q}}$ denotes the feature vector of node 𝑛 at the 𝑙-th layer of the GNN; the feature vector of neighbour node u at the (*l*-1)^th^ layer is represented by $\overset{\left(l-\text{1}\right)}{\underset{u}{x}}$; u∈N(n) represents the neighbour nodes 𝑢 that are in the set 𝑁(𝑛), which denotes the neighbourhood of node 𝑛. The expression for a mu*l*ti-layer GCN is as follows:


$$H^{\left(l+\text{1}\right)}=\sigma \left({\hat{C}}^{-\frac{\text{1}}{\text{2}}}\hat{A}{\hat{C}}^{-\frac{\text{1}}{\text{2}}}H^{\left(l\right)}w^{\left(l\right)}\right)$$ (2)

Where $\hat{A}=A+I$ and A is an adjacency matrix and I is the identity matrix; C and W are layered specific trainable weight matrices, (.)is the activation function, and the activation matrix in the (𝑙 + 1)th layer is denoted by $H^{\left(l+\text{1}\right)}$;$\;\hat{C}$ is a diagonal degree matrix of $\hat{A},$, i.e.,${\hat{C}}_{ii}=\in_{j}$, ${\hat{A}}_{ij}$.The convolution layers are roughly represented using Monte Carlo sampling if the graph can be split up into sub-graphs as follows:


$$H^{l+\text{1}}(v,:)=\sigma (\frac{\text{1}}{f_{l}}\sum_{i=\text{1}}^{f_{l}}\hat{A}\left(v,\overset{\left(l\right)}{\underset{i}{u}}\right)H\left(\overset{\left(l\right)}{\underset{i}{u}},:\right)W^{\left(l\right)})$$ (3)

where, for Monte Carlo samplings, $f_{l}$ is the number of independent and identically distributed samples $\overset{l}{\underset{\text{1}}{\;u}},\ldots ,\overset{l}{\underset{f_{\text{1}}}{u}}$; $H^{l+\text{1}}(v,:)$ is the feature vector of node *v at* layer *l +* 1; σ is the activation function;; $\hat{A}\left(v,\overset{\left(l\right)}{\underset{i}{u}}\right)$ is the normalized edge weight between node *v* and its neighbor $\overset{\left(l\right)}{\underset{i}{u}}$;$\;H\left(\overset{\left(l\right)}{\underset{i}{u}},:\right)$ is a feature vector of neighbour $\overset{\left(l\right)}{\underset{i}{u}}$ at layer *l*; W^*l*^ is learnable weight matrix at layer *l*. A basic illustration of a single convolution layer is as follows:


$$H=f\;(X,A)=\hat{A}ReLU\left(\hat{A}XW\right),$$ (4)

where H is output node embeddings and f(X,A) is a model’s learning condition; $\hat{A}\;$is a normalized adjacency matrix; X is a feature of the data, and ReLU represents the rectified linear unit non-linearity function; W is a learnable weight matrix, and A is the adjacency matrix. For understanding the architecture of a GCN model and the formation of GCN layers, the expression in (4) is helpful [35]. A GCN is formed by arranging several convolutional layers on top of one another.

#### Vision GCN.

Many studies that use graph neural networks for computer vision tasks are based on the standard graph convolutional network framework. However, this basic model is quite simple in both design and function. This, in turn, serves as the foundation for a more sophisticated model called the Vision Graph Convolutional Network. The Vision Graph Convolutional Network has several key components. First, all input images are pre-processed to ensure they are the same size. A lightweight stem module, consisting of multiple convolutional layers along with batch normalization, extracts basic visual features. These features are then enhanced with a positional embedding module that adds spatial information. At the center of the model is a backbone that alternates between a Deep Graph Convolutional Network and a feed-forward network, combined through random expansion aggregation. The Deep Graph Convolutional Network captures detailed structural patterns in the image using graph convolution operations. Meanwhile, the feed-forward network uses parameter sharing and DropPath regularization to improve efficiency and reduce overfitting. Together, these methods allow for stronger generalization by limiting parameter growth and managing complexity [36].

### Self-supervised learning

SSL is a novel paradigm in DL that learn high-quality data representations with minimal operator labelling [37]. SSL is classified into four categories: contrastive learning, clustering-based methods, generative learning, and predictive approaches. Contrastive learning is concerned with minimizing agreement between negative pairs and maximizing similarity among positive pairs. SimCLR and MoCo are used extensively as contrastive learning methods [38]. The methods, such as DeepCluster and SwAV, use cluster assignments as pseudo-labels to group unlabeled data into clusters, and they fall under Clustering-Based SSL [39]. Generative SSL methods like Auto-encoders and Variational Auto-encoders train an effective latent representation to reconstruct input data by using reconstruction loss functions like these:


$$L_{recon}={\left\|x-\hat{x}\right\|}^{\text{2}}$$ (5)

Where $L_{recon}$ is the reconstruction loss; *x* is the original input vector; $\hat{x}\;$is the output that has been recreated; ${\left\|x-\hat{x}\right\|}^{\text{2}}$ is the squared Euclidean distance [40]. Predictive methods such as BYOL and SimSiam attempt to match representations from different perspectives without the usage of negative samples by calculating the mean squared error between projected embeddings [41].

#### SimCLR.

**Fig 1:**
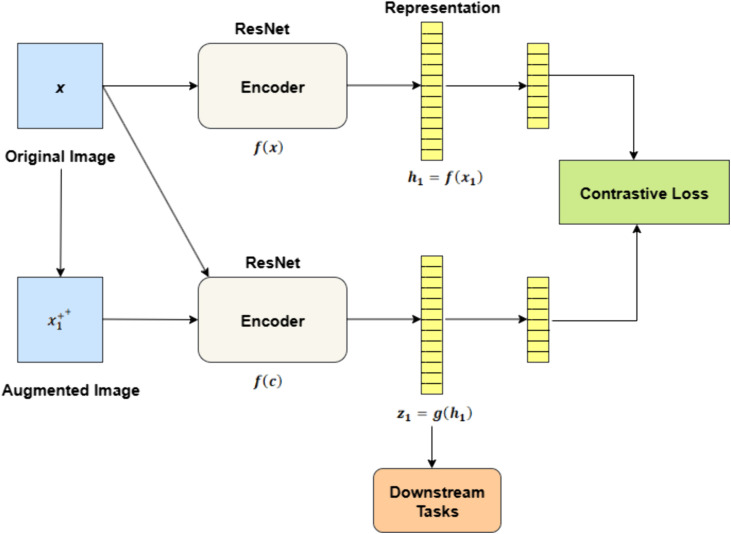
Contrastive loss calculation in SimCLR through ResNet based encoder network.


In SimCLR, a collection of N samples is first taken, and each of them is augmented in two different ways, which produces 2N variations in total. Rather than defining negative examples in advance, the framework treats every other augmented variation in the batch—apart from the chosen positive pair—as negatives, giving 2(N-1) implicit negative examples. The process by which contrastive loss is calculated is shown in Fig 1. Sim $(u,v)=u^{T}v/\left(\|u\|\|v\|\right)\;$is a representation of the cosine similarity between two instances u and *v.* The loss function of SimCLR for two positive cases (*i,j*) is defined as

$$L_{i,j}=-log\frac{exp\left(sim\left(z_{i},z_{j}\right)/T\right)}{\sum_{k=\text{1}}^{\text{2}N}{\text{1}}_{\left[k\neq \text{1}\right]}exp\left(sim\left(z_{i},z_{k}\right)/T\right)}$$ (6)

where ${\;L}_{i,j}$ is contrastive loss between a positive pair (*i,j);*$sim\left(z_{i},z_{j}\right)$ is similarity between embeddings $z_{i},z_{j}$; $z_{i}$ is embedding of the anchor sample and $z_{j}$ is embedding of the positive (matching) sample; T represent the temperature hyper-parameter;2N is total number of samples in the batch (N original examples × 2 augmentations); k is the index enumerating all embeddings in the batch, from 1 to 2N [42].

#### Deep Cluster.

**Fig 2:**
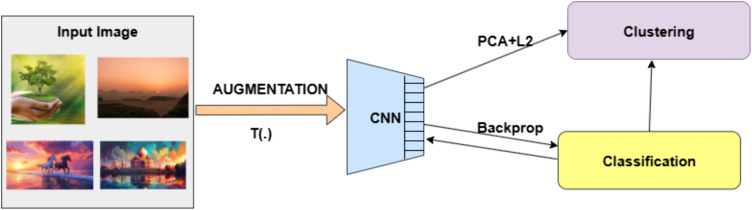
Deep cluster working methodology that depicts the clustering process through augmentation and back propagation.


Deep Cluster is a self-supervised learning method that combines clustering with representation learning in an iterative process. The working of the Deep cluster algorithm is shown in Fig 2. It functions by switching between two basic steps, each of which is symbolized by a significant optimization issue. The process first uses a common clustering algorithm, usually k-means, to group the features that the Convolutional Neural Network has extracted. Based on a geometric criterion, these features, represented as, $f_{\theta }\left(x_{n}\right)$ are grouped into k different groups. The goal of this step is to determine a centroid matrix $C^{*}$ and optimal cluster assignments $\left(y_{n}\right)n<N$. The optimization issue resolved in this case is:

$${min}_{{C\in R}^{d*k}}\frac{\text{1}}{N}\sum_{n=\text{1}}^{N}{min}_{y_{n}\in {\left\{\text{0},\text{1}\right\}}^{k}}\overset{\text{2}}{\underset{\text{2}}{\left\|f_{\theta }\left(x_{n}\right)-C_{y_{n}}\right\|}}\;such\;that\;\overset{T}{\underset{n}{y}}{\text{1}}_{k}=\text{1}$$ (7)

Here, ${C\in R}^{d*k}$ is matrix of cluster centroids, each column is a centroid; N is number of data samples;$\;x_{n}$ represents an input image; $f_{\theta }\left(x_{n}\right)\;$ is the feature extracted by the CNN with parameters θ; *C* is the centroid matrix; $y_{n}$ is the one-hot encoded cluster assignment for image $x_{n}$; $\overset{T}{\underset{n}{y}}{\text{1}}_{k}$ is constraint enforcing exactly one cluster assignment and k is number of cluster. The newly created cluster assignments, or pseudo-labels, are used as supervision in the second step to update the Convolutional Neural Network parameters. In simple terms, the model learns to map input to their designated clusters by being trained to predict these pseudo-labels. This is stated as a multinomial logistic loss function minimization problem:


$${min}_{\theta ,W}\frac{\text{1}}{N}\sum_{n=\text{1}}^{N}l\left(gw\left(f_{\theta }\left(x_{n}\right)\right),y_{n}\right)$$ (8)

Here, N is the number of training samples; *x*_*n*_ is the input sample n; *y*_*n*_ is the ground truth table for sample n; $f_{\theta }$ is the feature extractor parameterized θ;gw classifier head parameterized by w; *l* is the loss function [43].

#### BYOL.

**Fig 3:**
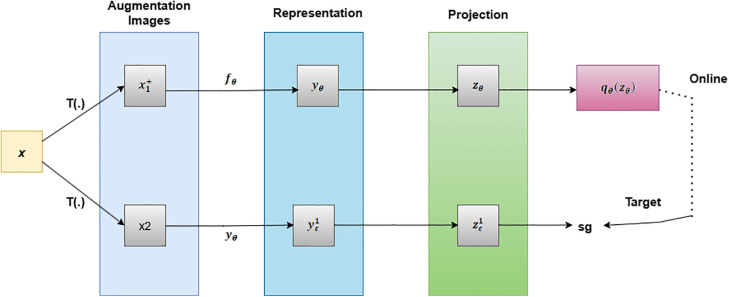
Working methodology of BYOL in target prediction through augmentation, representation and projection.


It learns visual representations without the usage of negative pairs, in contrast to traditional contrastive approaches. It has an online network and a target network as shown in Fig 3. They share the same architecture but differ in weight. The online network generates a prediction $q_{\theta }\left(z_{\theta }\right)$from *v* given two augmented views *v* and *v’* of the same image, whereas the target network generates a projection $\overset{\prime }{\underset{\varepsilon }{z}}$ from *v’*. The training goal is to reduce the mean squared error between these L2-normalized vectors, which is expressed as,

$$L_{\theta ,\varepsilon }={\left\|\frac{q_{\theta }\left(z_{\theta }\right)}{\|q_{\theta }\left(z_{\theta }\right)\|\text{2}}-\frac{\overset{\prime }{\underset{\varepsilon }{z}}}{\|\overset{\prime }{\underset{\varepsilon }{z}}\|\text{2}}\right\|}^{\text{2}}$$ (9)

Where $z_{\theta }$ is the Embedding of an augmented view of the input (from online encoder); $q_{\theta }(.)$ is the Predictor network applied to the online embedding; $\overset{\prime }{\underset{\varepsilon }{z}}$ is the embedding of another augmented view (from target encoder); ‖.‖ is the L2 norm (Euclidean norm). An exponential moving average of the online weights is used to update the target network weights ε:


 ε←Tε+(1−T)θ (10)

Where ε is the parameter of the target encoder;θ is the parameter of the online encoder; T is decay rate (hyperparameter) [41].

#### mRMR – Feature selection.

Working with high-dimensional data, such as histopathology images, requires careful feature selection. mRMR is a widely used filter-based approach for feature selection, designed to choose a subset of features that carry strong relevance to the target class while reducing overlap or redundancy among them [44]. Let S be the set of features that have been chosen, F be the full set of features, c be the target class, and *p(.)* be the probability. Mutual information is used to quantify a feature’s (f) relevance to the class.


$$I(f;c)=\sum_{f\in F}\sum_{c}p(f,c)log\frac{p(f,c)}{p(f)p(c)}$$ (11)

The mutual information *I*$\;\left(f_{i};f_{j}\right)\;$ is also used to calculate the redundancy among features $f_{i},f_{j}\in S.$. The objective function of mRMR is,


$$\text{max}S\;[\frac{\text{1}}{\left|S\right|}\sum_{f\in S}I(f;c)-\frac{\text{1}}{|S{|}^{\text{2}}}\sum_{f_{i,}f_{j}\in S}I\left(f_{i};f_{j}\right)\;]$$ (12)

Here |S| refers to the number of selected features. The trade-off between redundancy and relevance is balanced by this optimization. The initial term ensures that the selected features are instructive with respect to the target labels. The second term penalizes the selection of redundant or correlated characteristics.

## Methodology

**Fig 4:**
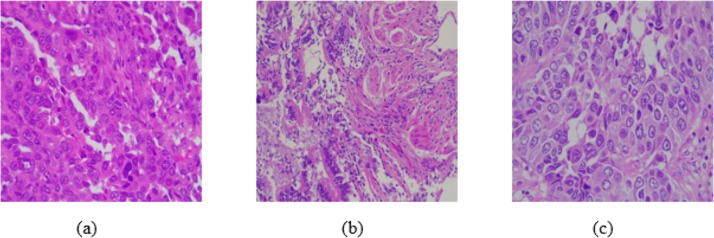
Sample Histopathological images from the LungHist700 dataset. a) Adenocarcinoma b) Normal c) Squamous Cell Carcinoma.

Experiments were majorly carried out using LungHist700 [45], a publicly available online dataset which includes 691 well-curated high-resolution histopathology images (1200 x 1600 pixels) obtained by digitization of 45 anonymized patients at 20x and 40x magnifications. It has three major classes: 280 Adenocarcinoma (ACC), 260 Squamous Cell Carcinoma (SCC), and 151 normal. Each image was extracted by expert pathologists from formalin-fixed, H&E-stained glass slides using a Leica microscope setup, ensuring clinical fidelity and heterogeneity reflective of real-world diagnostic variability. The sample histopathological images from the three classes are shown in Fig 4.

To validate the significant performance of the proposed model, two more datasets, namely LC25000 [46] and TCGA-UT [47], are used in this work. The LC25000 Dataset, curated by Andrew Mendez and made publicly available on Kaggle, serves as a significant resource for advancing automated cancer diagnosis through machine learning. It comprises 15,000 histopathological images collected from formalin-fixed paraffin-embedded tissue samples stained with hematoxylin and eosin. These images represent three major classes: lung adenocarcinoma, lung squamous cell carcinoma, and normal. Each image has a resolution of 768 × 768 pixels and was extracted from 86 whole-slide images acquired from patients treated at the Pathology Department of Hospital Universitario Virgen de la Victoria in Málaga, Spain. From this dataset, 250 images from each class are considered in this study. The TCGA-UT dataset, archived on Zenodo, represents a large-scale histopathology resource derived from hematoxylin and eosin-stained tumor slides across 32 solid cancer types from The Cancer Genome Atlas. Patches are standardized at 256 × 256 pixels. 500 histopathological patches, each from ACC and SCC classes, are considered in this study.

This study did not require new ethics approval from our institution as it is a secondary analysis of existing, publicly available, and de-identified datasets. The datasets were accessed for the purposes of this study between 15 May 2025 and 18 August 2025. The authors never had access to any participant identifiers or protected health information.

**Fig 5:**
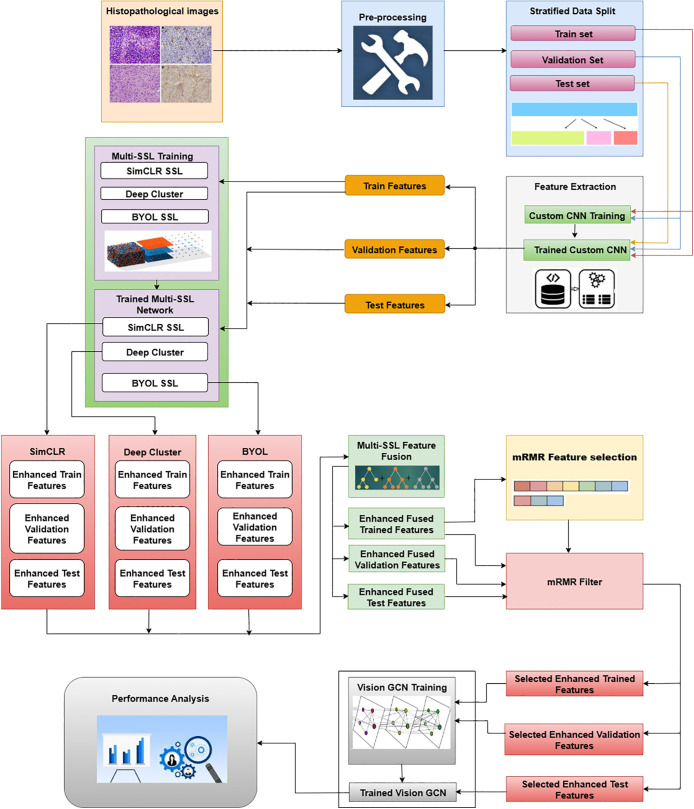
Overall Methodology that clearly shows the end-to-end pipeline of the proposed approach.

The proposed methodology combines Multi-Self Supervised Learning feature fusion with mRMR feature selection and Vision Graph Convolutional Network (MuSSL-mRMR-VGCN). The overall methodology is depicted in Fig 5. Initially, all the histopathological images belonging to three classes are pre-processed. The following steps are done during the pre-processing stage. Every image is normalized to the [0,1] range and resized to 224 x 224 pixels with a constant resolution to ensure consistency throughout training. In order to facilitate model compatibility, a Label Encoder is used to numerically encode the matching textual class labels. The dataset is divided into training, validation, and test subsets in the following proportions: 70%, 15%, and 15%, respectively, using stratified splitting. This guarantees that each split has a proportionate representation of each class. Data splitting was performed at the *patient level*. All patches or images originating from the same patient/slide were restricted to a single partition (train, validation, or test). This prevents any overlap between patient samples across different sets and eliminates the possibility of data leakage.

**Fig 6:**
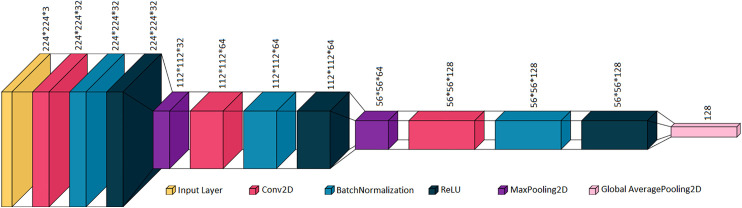
Custom CNN architecture for feature extraction that depicts the used layers and dimension of data processed at each layer.

After data splitting, a custom CNN architecture is designed and utilized for effective feature extraction from the pre-processed histopathological images. It is trained using training and validation sets, and the trained custom CNN extracts features from all three image sets – training, validation, and testing. From each histopathological image, 128 features are extracted. The architecture of the incorporated custom CNN is shown in Fig 6. The model takes an input image of size 224 × 224 × 3 and processes it through a sequence of convolutional layers. Initially, three stacked Conv2D layers with 32 filters each are applied, each followed by batch normalization and ReLU activation, preserving the spatial resolution while enriching local feature representations. A MaxPooling2D layer then reduces the spatial dimensions to 112 × 112 × 32. The previous step is followed by two convolutional layers with 64 filters, again using Batch Normalization and ReLU, and another MaxPooling2D layer further down samples the feature maps to 56 × 56 × 64. Batch-wise training is incorporated, and other model parameters such as batch size, learning rate, optimizer, etc., are presented in Table 1. Model parameter settings of different blocks are also shown in Table 1. In addition, the model configuration file is included in the supplementary section to document the hyperparameter settings and implementation details necessary for reproducing the experimental configuration.

**Table 1:** Model settings of various employed techniques.

Method	Parameter	Value/Details
Custom CNN	Batch Size	32
	Total Epochs	100
	Optimizer	Adam
	Learning Rate	0.001 (Search space: 1e-04,3e-04,5e-04, 1e-03, 3e-03)
	Loss Function	Cross-Entropy Loss
SSL Pre-training	Input Dim	128
	Hidden Dim	128
	Output Dim	128
	Optimizer	Adam
	Learning Rate	1e-3 (Search space: 1e-04,3e-04,5e-04, 1e-03, 3e-03)
	Epochs	100
	Augmentation	Gaussian noise (std = 0.05)
SimCLR	Loss	Normalized Temperature-Scaled Cross-Entropy
	Temperature	0.5 (Search space: 0.05, 0.1, 0.2, 0.5, 0.8, 1.0)
Deepcluster	Number of Clusters (k)	10 (Search space: 10, 20, 50)
	Criterion	Cross-Entropy Loss
	Pseudo-labeling	K-Means on encoder output
BYOL	Target Update Factor	0.99 (Exponential Moving Average)
	Loss	Mean Squared Error
Feature Selection	Selection Method	MIQ
	Number of Features	100 (Search space: 50–200)
Graph Construction & VGCN	Similarity Metric	Cosine similarity
	Dropout	0.4 (Search Space: 0.2,0.3,0.4,0.5)
	Loss Function	Focal Loss with α = 1, γ = 2 (Search space: α [0.25, 0.5, 1.0, 2.0], γ [0,1,2,3,4])
	Optimizer	Adam
	Epochs	100
	Early Stopping Patience	35
	Learning Rate	0.003(Search space: 1e-04,3e-04,5e-04, 1e-03, 3e-03)
	Top-K	15 (Search space: 5, 10, 15, 20, 25, 30)
	Evaluation Metric	Balanced Accuracy (BAC)

**Fig 7:**
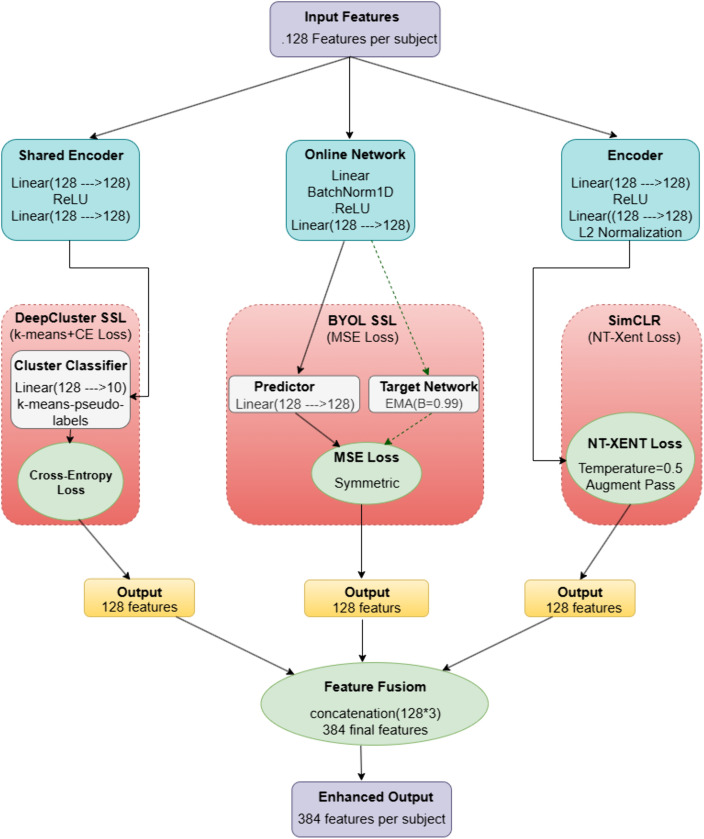
Deep feature optimization process that combines three different SSL techniques and generate fused features.

Then the extracted features are optimized using a fusion of three different SSL approaches as shown in Fig 7. Three SSL methods, namely SimCLR, DeepCluster, and BYOL, are employed for this operation to learn rich and complementary data representations. In the SimCLR branch, an encoder is incorporated. The encoder’s fundamental components are two linear layers with 128-dimensional input and output spaces, separated by ReLU activation and L2 normalization. This configuration makes features more appropriate for similarity-based learning by guaranteeing that they are projected into a structured embedding space with standardized magnitudes. The Normalized Temperature-Scaled Cross Entropy (NT-Xent) loss is utilized to find the similarity where augmented views of the same input sample are created and treated as positive pairs, whereas all other instances in the batch are treated as negative pairs. Using a temperature parameter (in this case set to 0.5) to regulate the concentration of the similarity distribution, the loss minimizes the distance between positive pairs while maximizing it for negative ones.

BYOL branch comprises an online network that projects features into a 128-dimensional latent space through a linear layer, batch normalization, ReLU activation, and another linear layer. The predicted embedding is produced by passing this representation through a predictor, which is a straightforward linear layer. A target network that has the same architecture as the online network simultaneously generates a target embedding. To ensure stability and consistency across training iterations, the target network is updated using an exponential moving average (EMA) of the online network’s weights rather than gradient descent. The momentum parameter is 𝛽 = 0.99. The online network is then encouraged to match its predictions with the gradually changing target representations by computing the Mean Squared Error (MSE) loss symmetrically between the predictor’s output and the target embedding. The Deep cluster branch projects the input features into a 128-dimensional latent space using a shared encoder made up of two linear layers divided by a ReLU activation. Following a linear layer and k-means clustering, these features are sent to a cluster classifier that divides them into a predetermined number of clusters. In our work, 10 clusters are considered. The classifier is then trained using a standard cross-entropy loss, using the cluster assignments that result as pseudo-labels.

During the training of all three SSL models, only the training set is utilized. After training, all three sets are passed as input to the trained model to get the enhanced 128 features individually. Then the output representations from each approach are concatenated to build an enriched feature embedding. After feature fusion, the mRMR algorithm is applied to the training features to determine which characteristics are the most relevant and least redundant, as the combined feature vectors may become high-dimensional. It selects the top 100 features from the 384 features. Following feature selection based on the training set, the selected 100 features are then consistently applied to the test and validation sets to preserve feature alignment. Next, graphs are built to incorporate VGCN. It uses cosine similarity matrices between each sample pair in the feature space, and each node (sample) is joined to its top-K most similar nodes to construct graphs. In the training graph, edge weights between samples of the same class are given a slight similarity boost to promote class cohesion.

**Fig 8:**
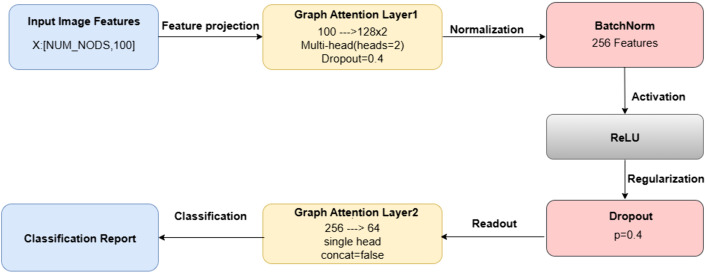
Structure of VGCN depicting two layers of Graph attention, activation function, normalization and regularization technique used.

Fig 8 shows the structure of VGCN incorporated. Graph Attention Layer 1 is the first step in the process. It uses a multi-head attention mechanism with two heads to convert 100-dimensional input features into a 128-dimensional space and applies dropout with a probability of 0.4 to avoid over-fitting. After normalizing the output across 256 features using Batch Normalization, a ReLU activation is applied to add non-linearity and improve the expressiveness of the model. To lessen neuronal co-adaptation, a subsequent Dropout layer with a 0.4 dropout rate serves as a regularization step. After processing, the features are sent to Graph Attention Layer 2, which reduces dimensionality from 256 to 64 by disabling feature concatenation and completing the final aggregation with a single attention head. This last layer creates concise and instructive node representations, acting as the readout stage for classification. In addition, Focal Loss, which focuses more on instances that are difficult to classify, is used as the training objective to address class imbalance. The model is trained using an Adam optimizer, and then a grid search is conducted across various learning rates and top-K values for graph formation. The detailed pseudo-code describing the proposed MuSSL–mRMR–VGCN pipeline is given in the supplementary materials section. All hyperparameter tuning was conducted exclusively on the validation set. The test set was held out and used only once for the final evaluation. This ensures that no information from the test set leaked into the model selection process. Early halting is applied based on validation accuracy to avoid over-fitting and save training time. Finally, the performance of the classifier is analysed using popular metrics, namely Balanced Accuracy (BAC) score, precision, recall, and F1-score.

## Results and discussion

**Fig 9:**
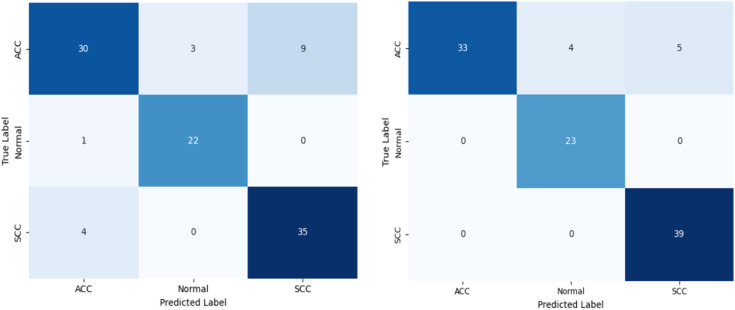
Class-wise confusion matrix of the plain and proposed classifiers (a) VGCN and (b) MuSSL  +  mRMR + VGCN. Three classes: Normal, ACC- Adenocarcinoma, SCC- Squamous Cell Carcinoma.

Initial experiments are made with a different set of feature extractors and classifiers. Feature extractors such as ResNET, EfficientNET, custom CNN, etc., are tested. K Nearest Neighbor, Random Forest, Support Vector Machine, Multilayer Perceptron, GNN, VGCN, etc., are tested as classifiers. Among them, custom CNN-based feature extraction and VGCN-based classification provided relatively better performance. Hence, for all the results provided in this section, a custom CNN is used as a feature extractor, and VGCN is used as a classifier. To improve the classification performance, feature optimization is attempted, and the proposed method MuSSL + mRMR+VGCN performed well. The confusion matrices of plain VGCN and the proposed method are shown in Fig 9. In the confusion matrix of plain VGCN, a few histopathological images from all three classes are misclassified, while only the images of ACC are misclassified in the proposed approach. Relatively, the number of ACC misclassifications is also less in the proposed approach.

Based on the confusion matrix, four different performance metrics are computed, namely, BAC, precision, recall, and F1 scores. BAC is primarily used since it can work well on the class-imbalanced dataset. Weighted average over three classes are considered for the calculation of precision, recall and F1 scores. Along with the plain and proposed methods, performance metrics of several other methods are presented in Table 2.

**Table 2:** Performance metrics obtained on Test data for the plain VGCN and different variants – LungHist700 dataset.

Methods	BAC	Precision	Recall	F1-Score
VGCN	0.86	0.84	0.84	0.83
simCLR+VGCN	0.77	0.72	0.7	0.68
Deepcluster+VGCN	0.78	0.62	0.57	0.46
BYOL+VGCN	0.85	0.8	0.78	0.77
MuSSL+VGCN	0.94	0.91	0.9	0.9
MuSSL + mRMR+VGCN	0.97	0.92	0.91	0.91
MuSSL+Lasso+VGCN	0.94	0.87	0.83	0.82
MuSSL + mRMR+Lasso+VGCN	0.93	0.91	0.91	0.91

As depicted in Table 2, the plain VGCN attains a BAC score of 0.86, and it can be seen that the usage of three SSL methods (simCLR, Deepcluster, BYOL) individually for feature enhancement leads to reduced BAC. In addition, precision, recall and F1 scores also reduce significantly. But MuSSL+VGCN, where the features from the same three methods are concatenated, provides relatively better performance than VGCN. This MuSSL+VGCN method offers the enhanced BAC of 0.94, and it supplies all 384 features (3 SSL methods * 128 features) to the VGCN. The reason for this better performance could be detailed as follows: When each SSL method is applied individually, the features learned tend to reflect only the strengths and biases of that specific approach, which can limit the diversity of information available to the VGCN and, in turn, reduce performance. In contrast, combining these feature sets in the MuSSL+VGCN framework brings together complementary aspects of representation: SimCLR’s emphasis on contrastive similarity, DeepCluster’s grouping-based semantics, and BYOL’s instance-level invariance. This fusion creates a more varied and information-rich feature space, allowing VGCN to better capture relationships and distinctions between classes, resulting in stronger predictive performance.

To improve the performance further, various filter-based and wrapper-based feature selection methods are experimented with to select the best features from the concatenated MuSSL features. Among the different wrapper-based feature selection methods tested, Lasso feature selection performed relatively well but failed to improve the performance, as it is capable of providing only the same BAC score of 0.94 as MuSSL+VGCN. Lasso applies L1 regularization to enforce sparsity, but in doing so, it may eliminate features that are weakly weighted yet still relevant, which limits performance improvement. On the other hand, mRMR provided significantly better performance among all the filter-based feature selection techniques. Hence, our proposed method is MuSSL + mRMR+VGCN, which offers the BAC score of 0.97. mRMR worked well because it explicitly selects features with high class relevance while minimizing redundancy, ensuring a more diverse and informative feature subset. The superior performance of MuSSL + mRMR+VGCN is thus the consequence of the best possible integration of VGCN for structural learning, mRMR for efficient feature selection, and MuSSL for rich representation. Hybridization of these two feature selection methods – mRMR & Lasso is also attempted, in which Lasso is used to select the top 200 features from the 384 features, and mRMR is used to select the top 100 features from the Lasso-selected 200 features. But this method also failed to provide enhanced performance. In the hybrid feature selection, the first stage (Lasso) reduces the feature space in a way that may already discard useful but subtle features, restricting mRMR’s ability to optimize selection and resulting in no performance gain.

DeepCluster, BYOL, and SimCLR each use unique algorithmic and architectural approaches that complement one another and this is another important reason for the better performance of the proposed model. For the purpose of differentiating histopathological subtypes, DeepCluster employs iterative clustering of feature embeddings to produce pseudo-labels that direct the network to capture global structural patterns and inter-class variations. BYOL is ideally suited to capturing subtle morphological differences because it uses a bootstrap mechanism with online and target networks to learn consistent and informative features without depending on negative samples. SimCLR emphasizes fine-grained local patterns by maximizing agreement between augmented views of the same image while separating different images through contrastive learning; however, it necessitates careful batch size and augmentation tuning. These techniques work well together for our downstream classification task in practice. SimCLR improves local detail discrimination, BYOL generates rich and stable embeddings, and DeepCluster offers coarse-grained clustering information. There are drawbacks to each approach as well: SimCLR is susceptible to batch size and augmentation, BYOL necessitates careful target network design, and DeepCluster can be computationally demanding. In our work, each framework’s unique strengths is capitalized by incorporating insights from all three into our multiple SSL pipeline. This enhances feature representation and classification performance overall while reducing the drawbacks of using just one method.

**Fig 10:**
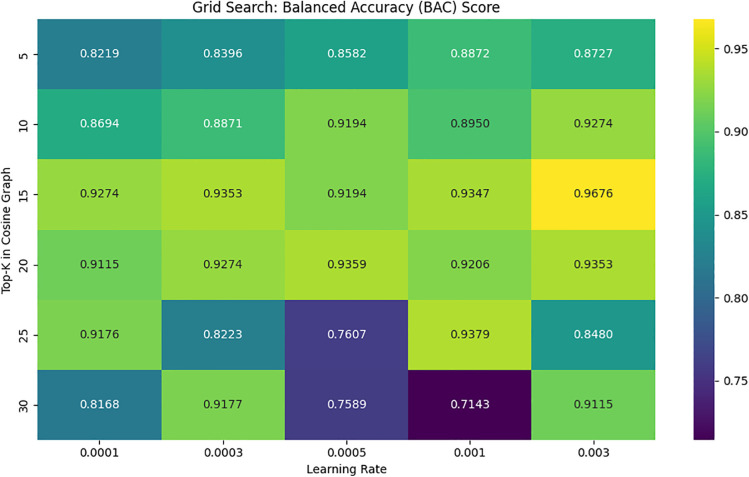
Hyper-parameter grid search for ideal Top-K value and learning rate in VGCN.

Another important reason for the enhanced classification performance is the proper fine-tuning of hyper-parameters. As mentioned in Table 1, ideal values of each hyper-parameter values are found through experimentation. Grid search is implemented in VGCN classifier during training based on the BAC score. Fig 10 shows the outcomes of a hyper-parameters grid search that was carried out to maximize the proposed model’s BAC score. The heatmap evaluates performance for various combinations of top-K values (y-axis) and learning rate (x-axis), where top-K is the number of neighbors implemented into consideration when building the cosine similarity-based graph used in the Vision GCN model. The BAC score obtained for a particular parameter setting is displayed in each grid cell. Performance is indicated by a color gradient, where higher accuracy is represented by brighter areas. Based on the findings, top-K = 15 and learning rate = 0.003 have the highest BAC score of 0.9676, suggesting that this is the best configuration among those examined. In the Heat-map, mid-range top-K values (10–20) consistently produce high BAC scores across a range of learning rates. However, using very low (learning rate = 0.001) and very high (top-K = 30) configurations results in lower BAC values, most likely as a result of over smoothing or under-fitting effects.

Table 3 provides the cross-validation results for all experimented models, serving to validate the performance presented in Table 2 and confirm that the observed results are not a random occurrence. Stratified 5-fold cross validation is used and, in this experiment, test set is exempted. Only the train & validation sets are combined, then cross validation is performed. Stratification ensures the presence of same proportion of classes in each fold. The results are clearly in-line with the results presented in Table 2. The proposed method achieved the highest BAC of 0.97 with a Standard Deviation (SD) of 0.01 across 5-folds. In addition, paired t-test, a popular statistical test is conducted to prove the better performance of the proposed model over other models. The obtained p-values of each model versus the proposed model is also presented in Table 3. While conducting the paired t-test, the identical sample folds are ensured in baseline model and proposed model for a fair comparison. Usually, if the p-value is less than 0.05, it can be considered as weak model compared to the proposed model. Plain VGCN offers the lowest p-value of 0.000046 while individual SSL methods also have very small p-value compared to the proposed model. This clearly suggest the improvements attained in the proposed model is not just due to random chance instead it is consistent. The p-value attained by two models namely, MuSSL+VGCN and MuSSL+Lasso+VGCN is above 0.05. Their p-values shows that statistically no significant difference was observed, suggesting that their performances are comparable to the proposed approach. Overall, the experimental results obtained from the 5-fold cross-validation demonstrate that the proposed method consistently outperforms the baseline models. The paired t-test analysis further confirms that the observed performance improvements are statistically significant (p < 0.05), indicating that the improvements are not due to random variation. The relatively low variance across folds also suggests that the proposed model maintains stable performance across different data splits.

**Table 3:** Performance metrics of Plain VGCN and its variants obtained on LungHist700 dataset through 5-fold cross validation.

Methods	BAC (Mean ± SD)	Precision (Mean ± SD)	Recall (Mean ± SD)	F1-Score (Mean ± SD)	Paired T Test (p-value)
VGCN	0.80 ± 0.05	0.80 ± 0.05	0.77 ± 0.07	0.76 ± 0.09	0.000046
simCLR+VGCN	0.73 ± 0.03	0.72 ± 0.03	0.70 ± 0.01	0.69 ± 0.01	0.000064
Deepcluster+ VGCN	0.71 ± 0.03	0.76 ± 0.02	0.70 ± 0.04	0.68 ± 0.06	0.000267
BYOL+VGCN	0.84 ± 0.04	0.84 ± 0.04	0.83 ± 0.04	0.83 ± 0.04	0.002174
MuSSL+VGCN	0.90 ± 0.04	0.91 ± 0.03	0.89 ± 0.05	0.89 ± 0.05	0.023837
MuSSL + mRMR+VGCN	0.97 ± 0.01	0.97 ± 0.01	0.97 ± 0.01	0.97 ± 0.01	–
MuSSL+Lasso+ VGCN	0.90 ± 0.04	0.91 ± 0.02	0.89 ± 0.03	0.89 ± 0.03	0.037580
MuSSL + mRMR+Lasso+VGCN	0.93 ± 0.02	0.92 ± 0.02	0.92 ± 0.02	0.92 ± 0.02	0.007757

**Fig 11:**
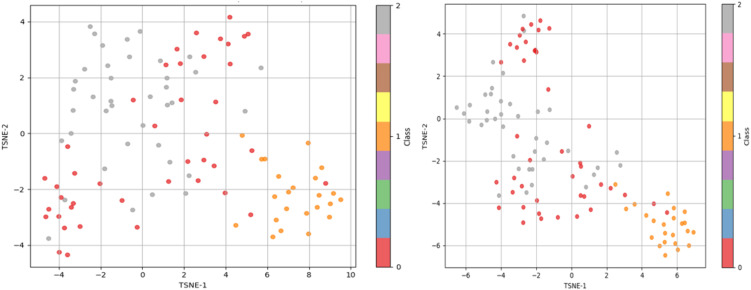
t-SNE plots to visualize feature optimization provided by MuSSL + mRMR (a) t-SNE plot of Original Features; (b) t-SNE plot of MuSSL + mRMR optimized features.

Next, the feature optimization through the proposed MuSSL + mRMR-VGCN pipeline is demonstrated through the t-distributed Stochastic Neighbour Embedding (t-SNE) graphs between the original features and MuSSL + mRMR enhanced features. It is presented in Fig 11. In particular, class separability can be visually assessed using these plots, which project high-dimensional feature representations of histopathological lung images into two-dimensional space. Fig 11 (a) plot presents the raw feature distribution before any feature enhancement, and these are the original features. Although there is certain visual clustering, the classes normal, ACC, and SCC overlap significantly. The existence of overlapping regions shows that the original feature set is redundant and full of noise, which can interfere with the performance of classification.Fig 11(b) demonstrates the features after MuSSL + mRMR. In this case, the clusters are relatively clear and further apart, with a better differentiation of the three tissue classes. This demonstrates the capability of the proposed method in feature optimization to enhance class separability.

The class-wise performance metrics will give more clarity regarding the performance of classifiers. Hence, the class-wise metrics are presented in Table 4 for all the methods investigated. VGCN offers moderate performance with F1 scores of 0.78, 0.92, and 0.84 for the three classes ACC, Normal, and SCC, respectively. Relatively, VGCN is good at predicting the normal class and poor at predicting the ACC class. The same scenario happens in all the methods investigated, where correctly predicting the ACC class is difficult and predicting the normal class is easier. The lower performance in classifying ACC compared to normal cases may arise from higher morphological diversity or subtle feature differences in ACC tumors, making them harder to distinguish from the more homogeneous normal tissue. In contrast, normal samples typically exhibit consistent anatomical patterns, leading to higher detection rates. When precision and recall of various models are compared, almost precision is relatively high while classifying the ACC cases, and the Recall score is relatively high while classifying Normal and SCC cases. The higher precision for ACC cases suggests the model correctly identifies true ACC samples with minimal false positives, while the higher recall for Normal/SCC cases indicates fewer missed detections, likely due to their more distinct or consistent features compared to ACC’s variability.

**Table 4:** Class-wise Performance Metrics Comparison.

Methods	Class	Precision	Recall	F1-score
VGCN	ACC	0.86	0.71	0.78
	Normal	0.88	0.96	0.92
	SCC	0.8	0.9	0.84
SimCLR+VGCN	ACC	0.74	0.4	0.52
	Normal	0.88	0.91	0.89
	SCC	0.61	0.9	0.73
Deepcluster+VGCN	ACC	0.67	0.05	0.09
	Normal	0.75	0.91	0.82
	SCC	0.49	0.92	0.64
BYOL+VGCN	ACC	0.88	0.55	0.68
	Normal	0.85	0.96	0.9
	SCC	0.69	0.92	0.79
MuSSL+VGCN	ACC	0.94	0.81	0.87
	Normal	0.82	1	0.9
	SCC	0.93	0.95	0.94
MuSSL + mRMR+VGCN	ACC	1	0.79	0.88
	Normal	0.85	1	0.92
	SCC	0.89	1	0.94
MuSSL+Lasso+VGCN	ACC	1	0.57	0.73
	Normal	0.88	1	0.94
	SCC	0.72	1	0.84
MuSSL + mRMR+lasso+VGCN	ACC	0.9	0.88	0.89
	Normal	0.92	1	0.96
	SCC	0.92	0.9	0.91

When compared to VGCN, the three individual SSL models provide poor performance, and the same can be witnessed in Table 4. But the concatenation of three SSL methods yields a significant rise in class-wise metrics. Particularly, the proposed model, MuSSL + mRMR+ VGCN performed well in providing better precision, recall, and F1 score for all three classes. The poor performance of individual SSL models likely stems from their limited ability to capture the diverse feature representations needed for accurate classification. However, concatenating three SSL methods compensates for their individual weaknesses by combining complementary feature spaces, leading to more robust discrimination. The superior performance of MuSSL + mRMR+VGCN can be attributed to its hybrid approach – leveraging multi-view SSL for richer feature learning, mRMR for optimal feature selection, and VGCN for effective graph-based classification – resulting in enhanced precision, recall, and F1 scores across all classes.

The proposed framework demonstrated differential predictive performance across lung cancer subtypes as shown in Table 4. The F1-score for squamous cell carcinoma was 0.94, whereas adenocarcinoma achieved 0.88, indicating that the model classified squamous cell carcinoma more accurately and consistently. This performance gap suggests that the Vision-GCN model more effectively captured the spatial and morphological cues characteristic of squamous lesions. The underlying reason for this disparity lies in the distinct histopathological characteristics of these subtypes. Squamous cell carcinoma typically presents well-defined keratinization, polygonal cell morphology, and strong intercellular junctions, producing clearer structural boundaries and less intra-class variation. These stable spatial features align well with the graph-based modeling in the Vision-GCN, enabling the network to learn discriminative representations efficiently. Conversely, adenocarcinoma is characterized by heterogeneous glandular architecture, irregular nuclear morphology, mucinous cytoplasm, and variable tissue density, leading to greater intra-class variability and overlapping feature distributions. Such heterogeneity often reduces the feature separability of adenocarcinoma, making accurate classification more challenging. Overall, while the MuSSL + mRMR + VGCN framework generalizes effectively across subtypes, its performance on adenocarcinoma could be enhanced through subtype-specific data augmentation, balanced sampling, and fine-tuning of self-supervised feature representations to better capture its complex tissue organization and variability.

Computation time taken for executing various methods is presented in Table 5. Among them, custom CNN takes a very high time since its training takes more time, and it is crucial for appropriate feature extraction. Then the extracted features are given as input to various models.

**Table 5:** Computation Time across various methods.

Method	Computation Time(hh:mm:ss)
Custom CNN	02:11:20
VGCN	00:01:18
SimCLR+VGCN	00:01:50
Deepcluster+VGCN	00:02:08
BYOL+VGCN	00:01:21
MuSSL+VGCN	00:04:48
MuSSL + mRMR+VGCN	00:07:41

The usage of individual SSL slightly increases the computation time when compared to plain VGCN. Among the three methods, BYOL+VGCN is the fastest, and Deepcluster+VGCN takes relatively more time to execute. The increased computation time of individual SSL methods compared to plain VGCN arises from their additional pre-training or contrastive learning overhead. BYOL+VGCN’s faster execution likely stems from its simpler negative-sample-free architecture, while DeepCluster+VGCN’s slower runtime reflects its iterative clustering steps and higher feature-processing demands. The fusion of three SSL methods, MuSSL+VGCN and the proposed method, MuSSL + mRMR+VGCN, obviously takes relatively higher computation time than the plain VGCN and individual SSL-based methods.

As mentioned in the methodology section, all the models which are investigated on the first dataset – LungHist700 are validated with two more of above mentioned datasets. The same methodology is employed, and the crucial performance metrics, namely BAC, weighted average scores- precision, recall, and F1 scores on these two datasets are presented in Table 6. Unlike the first dataset, a good balance between precision and recall scores can be seen on the other two datasets for all the models investigated, and this can be witnessed in Table 6. The improved balance between precision and recall in the other two datasets suggests these datasets likely have more distinct class distributions or fewer ambiguous samples, allowing the model to achieve better harmony in identifying positives (recall) while minimizing false alarms (precision). This alignment naturally leads to higher F1 scores, which depend on both metrics, and correlates well with the BAC that equally weights all classes.

**Table 6:** Performance metrics of plain VGCN and different variants on the other two datasets.

Dataset	Methods	BAC	Precision	Recall	F1-score
Dataset-2	VGCN	0.95	0.95	0.95	0.95
	simCLR+VGCN	0.89	0.84	0.84	0.83
	Deepcluster+VGCN	0.92	0.92	0.92	0.92
	BYOL+VGCN	0.94	0.89	0.87	0.86
	MuSSL+VGCN	0.94	0.92	0.92	0.92
	MuSSL + mRMR+VGCN	1	0.99	0.99	0.99
	MuSSL+Lasso+VGCN	0.96	0.9	0.89	0.89
	MuSSL + mRMR+Lasso+VGCN	0.97	0.96	0.96	0.96
Dataset-3	VGCN	0.95	0.93	0.93	0.93
	simCLR+VGCN	0.74	0.73	0.73	0.73
	Deepcluster+VGCN	0.87	0.83	0.79	0.78
	BYOL+VGCN	0.94	0.94	0.94	0.94
	MuSSL+VGCN	0.93	0.95	0.95	0.95
	MuSSL + mRMR+VGCN	0.99	0.99	0.99	0.99
	MuSSL+Lasso+VGCN	0.98	0.94	0.93	0.93
	MuSSL + mRMR+Lasso+VGCN	0.99	0.99	0.99	0.99

**Fig 12:**
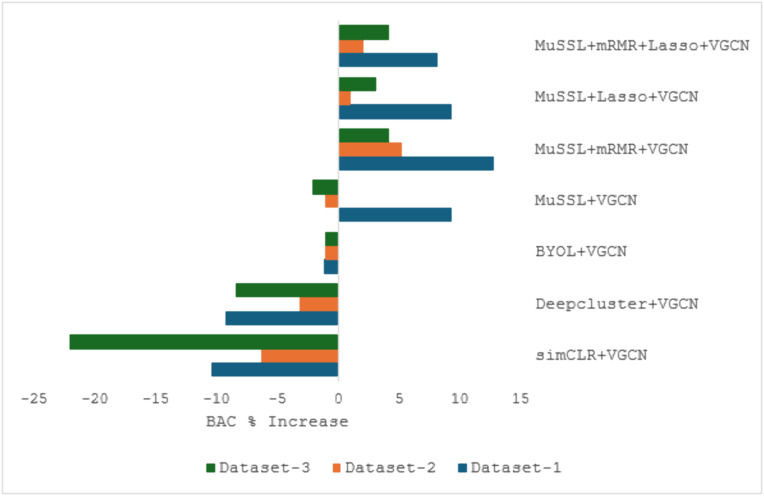
Comparison chart depicting the BAC% increase offered by various models when compared to plain VGCN across all the three datasets.

To understand the significance of the proposed method further, the increase in BAC% when compared to plain VGCN is plotted for all three datasets in Fig 12. The plain VGCN offers 0.95 BAC in both the Dataset-2 and Dataset-3. Similar to the results obtained from the first dataset, performance is degraded if individual SSL methods are used for feature optimization when compared to plain VGCN. This can be well interpreted from Fig 12, where a negative BAC% increase is witnessed for all three datasets. The performance degradation with individual SSL methods suggests that their feature optimization may introduce noise or redundancy, disrupting VGCN’s inherent discriminative power.

Unlike the first dataset, the model based on the fusion of three SSL methods – MuSSL+VGCN not work well in the other two datasets. Slightly BAC is reduced in this model when compared to the plain VGCN. The reduced BAC of MuSSL+VGCN in Datasets 2 and 3 suggests that the fused SSL features may not generalize as effectively across diverse data distributions, possibly due to dataset-specific biases, and so feature selection becomes the key. The usage of both filter and wrapper-based feature selection techniques yielded relatively better performance than plain VGCN, and this shows the importance of feature selection on the fused SSL optimized features. The improved performance with filter and wrapper-based feature selection highlights that fused SSL features contain redundant or noisy dimensions, possibly due to dataset-specific biases, which degrade model performance if left unrefined. By selecting only the most discriminative features, these techniques enhance VGCN’s ability to leverage optimized representations, proving that effective feature selection is critical for maximizing SSL-augmented learning.

Similar to the first dataset, the proposed MuSSL + mRMR+VGCN method worked well and offered the highest BAC and weighted average scores on the other two datasets. It is capable of producing 5.2% and 4.2% BAC increase when compared to plain VGCN on the other two datasets. Though the MuSSL+Lasso+VGCN and MuSSL + mRMR+Lasso+VGCN offered positive BAC% increase, they are not able to beat the proposed MuSSL + mRMR+VGCN approach in all three experimented datasets. The superior performance of MuSSL + mRMR+VGCN can be attributed to mRMR’s ability to retain discriminative yet non-redundant features from the fused SSL representations, while Lasso-based methods may over-penalize useful features due to their aggressive sparsity constraints. This optimal balance between relevance and redundancy in mRMR enables the model to generalize robustly across all datasets, unlike Lasso-inclusive variants, which sacrifice critical information for simplicity. Despite the promising results achieved across multiple datasets, certain limitations should be acknowledged regarding the generalizability of the proposed framework. The datasets used in this study may not fully capture the diversity encountered in real-world clinical environments, where variations in scanner types, staining protocols, slide preparation procedures, and patient demographics may introduce domain shifts. Addressing these challenges requires further validation on larger multi-institutional datasets and the development of techniques such as stain normalization, domain adaptation, and robust representation learning to improve cross-domain generalization.

The accuracy attained in various works related to lung cancer classification is summarized in Table 7 and compared with the proposed method. It clearly shows the relatively better performance of the proposed method when compared to a few other works in the literature.

**Table 7:** Accuracy comparison of related works.

Related work	Year	Classification framework	Accuracy (%)
Jain et al. [2]	2024	Ensemble CNN + reinforcement learning	92
Ram et al. [27]	2023	Graph-based sparse PCA with SVM	92
Coudray, N.et al. [12]	2018	Deep CNN on histology slide	91
Nissar, A. et.al. [48]	2024	Radiomics (Shape + WPT) – MGSjVM model	90
Dunn, B. et.al. [49]	2023	Radiomics classifiers	88
Shafi et.al. [50]	2022	Hybrid SVM + CNN model	94
Xu, Hua.et al. [51]	2024	VGG16-based CNN	92
Sohaib. et al. [52]	2023	Risk-factor deep learning model	94
Pan, Z., Hu, G. et.al. [53]	2024	Ternary DL model	87
Proposed	2025	MuSSL + mRMR+VGCN	97

The intrinsic characteristics and biases of the LungHist700, LC25000, and TCGA-UT datasets exert distinct yet interrelated influences on the external validity of models trained upon them. LungHist700, while curated and balanced, suffers from limited institutional diversity, which constrains its applicability to broader clinical settings. LC25000 offers scale and accessibility but introduces biases through patch-based sampling and synthetic augmentation, potentially inflating benchmark performance while failing to capture real-world histopathological variability. TCGA-UT provides greater heterogeneity and demographic representation yet exhibits domain shift challenges due to variable slide preparation protocols and class imbalance. Collectively, these factors highlight the risk of overfitting to dataset-specific artifacts and underscore the necessity for careful validation when extrapolating findings to external populations. Biases may be quantitatively assessed through cross-dataset testing, distributional shift measures, and subgroup-specific performance analysis, while qualitative evaluations can include expert pathology review and representation visualization. To mitigate these limitations, future research should prioritize multi-institutional data integration, stain normalization, and redundancy reduction, particularly for patch-based datasets.

### Future directions.

Future studies should concentrate on improving the suggested multiple self-supervised learning framework for lung cancer histopathology image classification in terms of its effectiveness, resilience, and clinical relevance. Creating lightweight fusion techniques, like knowledge distillation or model pruning, can lower processing demands and make deployment easier in environments with constrained resources. To guarantee generalizability in a variety of clinical contexts, the model should also be verified on multi-center datasets with various imaging protocols and staining variations. Integration into diagnostic workflows can be facilitated by enhancing interpretability by associating important features with recognized histopathological patterns. Important directions also include evaluating cross-dataset generalization, investigating hybrid SSL and semi-supervised methodologies, incorporating multi-modal data, and applying explainable AI techniques to offer feature-level insights.

To transition this research into a clinically viable tool for early lung cancer detection, several critical steps are required. First, rigorous validation on diverse, multi-institutional datasets and prospective clinical trials is essential to ensure external validity and generalizability beyond retrospective analyses. Second, integration into clinical practice necessitates explainability mechanisms and seamless incorporation into radiology and pathology workflows, positioning the system as an assistive decision-support tool. Third, regulatory approval requires comprehensive evidence of safety, efficacy, and reproducibility, alongside careful audits of demographic fairness and strict adherence to data privacy frameworks. Finally, robust technical deployment demands optimization for efficiency, resilience to domain shifts, and ongoing monitoring to maintain performance in real-world settings. Collectively, these steps may provide a pathway to translating algorithmic advances into practical, trustworthy solutions for early lung cancer detection.

## Conclusion

In this work, we proposed a novel effective framework for the classification of histopathology lung images that combines Multi-Self Supervised Learning, Minimum Redundancy Maximum Relevance feature selection, and Vision Graph Convolutional Networks. The proposed MuSSL + mRMR+VGCN model offered a BAC of 97% and an increase of 12% BAC when compared to plain VGCN on the LungHist700 dataset. The superior performance of the proposed method is validated with two more publicly available datasets. The MuSSL+VGCN framework outperforms individual SSL methods by combining their complementary representations - SimCLR’s contrastive learning, DeepCluster’s semantic grouping, and BYOL’s instance invariance. The superior performance of MuSSL + mRMR+VGCN is thus the consequence of the best possible integration of VGCN for structural learning, mRMR for efficient feature selection, and MuSSL for rich feature representation. While the proposed MuSSL + mRMR+VGCN framework demonstrates superior performance, its computational complexity may hinder real-time deployment, and its generalizability could be limited by dataset-specific biases such as staining variations or rare cancer subtypes. Future efforts should focus on developing lightweight fusion techniques (e.g., knowledge distillation) to optimize efficiency, validating the model on multi-center datasets with diverse imaging protocols to enhance robustness, and improving interpretability by linking selected features to clinically relevant histopathological patterns for better integration into diagnostic workflows.

## Supporting information

S1 File Model configuration file and Pseudocode of the proposed model MuSSL + mRMR+VGCN. (ZIP)
