# Supplementary material for: Deep feature optimization using fusion of multiple self-supervised learning approaches and filter-based feature selection for lung cancer histopathology classification
Source: PLoS One. 2026 Apr 27;21(4):e0348194. doi: 10.1371/journal.pone.0348194 (PMC13119965; doi:10.1371/journal.pone.0348194)
Supplement: S1 File — (ZIP) [file pone.0348194.s001.zip › Pseudocode of the proposed model MuSSL.pdf]

Pseudocode of the proposed model MuSSL+mRMR+ VGCN:

Input:
- Image dataset with classes {acc, scc, normal}
- Train/Validation/Test split ratios: 0.15 test, 0.15 validation
- Random seed = 42
- Image size = 224 × 224

Output:
- Classification report (mean ± std) from stratified 5-fold CV
- Classification report on final test set
- Trained VisionGNN model

Step 1: Load and preprocess images

----

1. Initialize random seeds.
2. For each class {acc, scc, normal}:
   Load all images → RGB → resize 224×224 → normalize.
   Store images and labels.
3. Encode labels to integer classes.
4. Split dataset into:
   • 15% test set
   • Remaining → train and validation (17.5% of remainder as validation)

Step 2: CNN-based feature extraction

----

5. Define SimpleCNN:
   Conv(3→32)→BN→ReLU→MaxPool(2)
   Conv(32→64)→BN→ReLU→MaxPool(2)
   Conv(64→128)→BN→ReLU→AdaptiveAvgPool(1)
6. Attach FC layer Linear(128→3) for training.
7. Train classifier for 100 epochs:
   Loss = CrossEntropyLoss
   Optimizer = Adam(lr=0.001)
   Batch size = 32
8. Remove classifier head and retain CNN backbone.
9. Extract 128-dim CNN features for train, val, test.
10. Save all extracted features to CSV.

Step 3: Load extracted features

----

11. Load train_feats, val_feats, test_feats as float tensors.

Step 4: Self-Supervised Feature Learning (SSL)

----------- (4A) Contrastive Learning -----------

12. Initialize encoder: MLP(128→128).
13. For 100 epochs:
    - Generate two Gaussian-noise views per sample (std=0.05)
    - Compute NT-Xent loss (temperature=0.5)
    - Optimize with Adam(lr=1e-3)
14. Save encoder.

----------- (4B) DeepCluster -----------

15. Initialize encoder + classifier (10 clusters).
16. Compute initial features → run KMeans clustering (k=10).
17. Use pseudo-labels for training.
18. For 100 epochs:
    Minimize CrossEntropyLoss over pseudo-labels (lr=1e-3).
19. Save encoder only.

----------- (4C) BYOL -----------

20. Initialize BYOL (online, target, predictor MLPs).
21. For 100 epochs:
    - Generate two Gaussian-noise views
    - Compute BYOL loss
    - Optimize online network using Adam (lr=1e-3)
    - Update target by EMA with beta=0.99
22. Save online encoder.

Step 5: Feature merging + mRMR

---

23. Compute SSL embeddings separately for train, val, test for:
    - Contrastive
    - DeepCluster
    - BYOL
24. Concatenate the three embeddings into merged feature vectors.
25. Create DataFrame with train features + labels.
26. Apply mRMR(MIQ) to select top 100 features.
27. Reduce train, val, test features to these selected 100.

Step 6: Graph construction

---

28. For each split (train, val, test):
    - Compute cosine similarity matrix S
    - Zero out diagonals
    - If same label, boost similarity by +0.2

• Select top_k strongest edges per node
• Construct graph:
  x = selected features
  y = labels
  edge_index = selected edges

Step 7: VisionGNN architecture
----
29. Define VisionGNN:
  Layer 1: GATConv(in_dim → 64, heads=2, dropout=0.4), BatchNorm, ReLU + Dropout(0.4)
  Layer 2: GATConv(64*2 → 3, heads=1, concat=False)

30. Loss function: FocalLoss(alpha=1, gamma=2)

Step 8: Stratified 5-Fold Cross-Validation
----
31. Split training data into 5 stratified folds.
32. For each fold:
  a. Build graph using training folds.
  b. Build validation graph using held-out fold.
  c. Train VisionGNN for up to 100 epochs:
    - Optimizer: Adam(lr=1e-3)
    - Early stopping patience = 35
  d. Evaluate fold model on validation graph.
  e. Compute fold Balanced Accuracy (BAC).
33. Compute final metrics:
  Mean score = average of 5 folds
  Std score = standard deviation of 5 folds

Step 9: Grid search for best Top-K and Learning Rate
----
34. For each top_k ∈ {5,10,15,20,25,30}:
  For each lr ∈ {1e-4, 3e-4, 5e-4, 1e-3, 3e-3}:
    • Build train, val, test graphs with top_k
    • Train VisionGNN with Adam(lr) for 100 epochs
    • Early stop when patience = 35
    • Evaluate best model on test graph
    • Record BAC
35. Select the configuration (top_k*, lr*) with highest BAC.

Step 10: Final training on full training data
----
36. Rebuild graphs using top_k*.
37. Retrain VisionGNN using lr* with early stopping.

38. Save best model weights.

Step 11: Final evaluation on test data

----

39. Load best model state.
40. Predict class labels for test graph.
41. Generate full classification report (precision, recall, F1, accuracy).
42. Return:
    - Mean ± Std Balanced Accuracy from 5-Fold CV
    - Final test classification report
    - Trained VisionGNN model

End
